# How Hydrodynamic Phonon Transport Determines the Convergence of Thermal Conductivity in Two-Dimensional Materials

**DOI:** 10.3390/nano12162854

**Published:** 2022-08-18

**Authors:** Jianhui Jiang, Shuang Lu, Yulou Ouyang, Jie Chen

**Affiliations:** Center for Phononics and Thermal Energy Science, China–EU Joint Lab for Nanophononics, MOE Key Laboratory of Advanced Micro-structured Materials, School of Physics Science and Engineering, Tongji University, Shanghai 200092, China

**Keywords:** thermal conductivity, two-dimensional materials, hydrodynamic phonon transport, convergence

## Abstract

The phonon Boltzmann transport equation combined with first-principles calculation has achieved great success in exploring the lattice thermal conductivity (κ) of various materials. However, the convergence of the predicted κ is a critical issue, leading to quite scattered results recorded in the literature, even for the same material. In this paper, we explore the origin for the convergence of thermal conductivity in two-dimensional (2D) materials. Two kinds of typical 2D materials, graphene and silicene, are studied, and the bulk silicon is also compared as a control system for a three-dimensional material. The effect of the cutoff radius ($r_{c}$) in the third-order interatomic force constants on κ is studied for these three materials. It is found that that κ of these three materials exhibits diverse convergence behaviors with respect to $r_{c}$, which coincides very well with the strength of hydrodynamic phonon transport. By further analyzing the phonon lifetime and scattering rates, we reveal that the dominance of the normal scattering process gives rise to the hydrodynamic phonon transport in both graphene and silicene, which results in long-range interaction and a large lifetime of low-frequency flexural acoustic phonons, while the same phenomenon is absent in bulk silicon. Our study highlights the importance of long-range interaction associated with hydrodynamic phonon transport in determining the thermal conductivity of 2D materials.

## 1. Introduction

As the size of novel electronic devices gradually shrinks to the nanoscale, thermal management and energy conversion at such length scale are facing huge challenges [1,2,3]. The diverse thermophysical properties make the two-dimensional (2D) materials suitable candidates for both heat dissipation [4,5,6,7,8] and thermoelectric applications [9,10,11]. Understanding the phonon thermal transport in 2D materials [12] is very important for designing new 2D materials and developing their applications. Important factors affecting the thermal transport properties of solids have been revealed, such as the phonon coherence [13,14,15,16,17], symmetry [18,19], four-phonon scattering [20,21,22,23], system size [24], and hydrodynamic phonon transport [25,26,27,28,29,30].

The phonon Boltzmann transport equation (BTE) is a powerful tool to predict the material’s lattice thermal conductivity (κ) and understand the underlying phonon transport mechanism [31,32,33,34,35,36,37,38]. Its prediction accuracy depends on a number of input parameters [39,40,41,42,43]. Among them, the cutoff radius ($r_{c}$) for the nearest neighbors’ (NNs) interaction is critical in predicting the κ of 2D materials. For instance, Qin et al. [40] found that the predicted κ values for graphene by BTE are quite different with different $r_{c}$, and a large $r_{c}$ is required to ensure the convergence of κ. Similarly, Sun et al. [44] found in four kinds of stable 2D honeycomb structures that the predicted value of κ varies greatly with different $r_{c}$, which can differ by a factor of 2 if an insufficiently large $r_{c}$ is used, and a large $r_{c}$ up to the 14^th^ NN should be used to reach convergence. In contrast, the predicted κ of three-dimensional (3D) materials is less sensitive to the choice of $r_{c}$ [45,46].

Possible origins for the convergence issue for κ of 2D materials have been identified. One is that the existence of long-range interatomic forces [40,47], such as resonant bonds, which were first found in rock-salt-like crystal structures, and the decoupling of the π-bond. Another is that the truncation errors in existing calculations can lead to the divergence of κ. Lindsay et al. [48] reported that a small supercell or a finite number of nearest neighbor atomic layers may break the principle of the translational invariance (TI) condition of the crystal. Similarly, Taheri et al. [35] found that the hybridization of flexural acoustic (ZA) modes and transverse/longitudinal acoustic (TA/LA) modes in 2D materials, as well as the limitation of the supercell size could break the rotational symmetry in second-order interatomic force constants (IFCs). However, the supercell used in previous works [40,44,49] is not sufficiently large, so that the $r_{c}$ for the NNs’ interaction exceeds half of the supercell length, which can greatly affect the prediction accuracy. Moreover, the influence of the NNs in the third-order IFCs, as well as the different sensitivity to the NNs for different dimensional systems are still unknow.

In this work, we investigate the relationship between κ and $r_{c}$ of the third-order IFCs in different materials, including graphene, silicene, and silicon. By calculating the value of κ with different $r_{c}$, we find that the κ of graphene is the most difficult one to converge as $r_{c}$ increases, while silicon’s κ is not sensitive to the NNs and can converge within a small $r_{c}$. Through the study of phonon–phonon scattering process, we discovered that the dominant normal scattering enables long-range interatomic force in hydrodynamic 2D materials, such as graphene. By further analysis of each phonon mode, we found that the underlying physical mechanism lies in the strong anharmonic ZA phonons, while TA and LA are insensitive to $r_{c}$. Our work provides physical insights into the deeper understanding of heat transport and hydrodynamic phonon transport in different systems.

## 2. Computational Method

All first-principles calculations were performed based on density functional theory (DFT), as implemented in the Vienna ab initio Simulation Package (VASP) [50,51,52] with the projected augmented wave method. The Perdew–Burke–Ernzerhof (PBE) of generalized gradient approximation (GGA) was used as the exchange-correlation functional in our study, since it has already been shown to have an accurate description of a smoother potential, correct behavior under uniform scaling, and a linear response of the uniform electron gas [53]. A vacuum space of 20 Å was imposed along the c-axis only for 2D materials to exclude the interaction between adjacent imaging layers. In all three structures, the unit cell was optimized with a cutoff energy of 600 eV until the energy and the Hellmann–Feynman force converged to 10^−8^ eV and 10^−4^ eV ${Å}^{-1}$, respectively. Besides, the k-mesh grid used to sample the Brillouin zone (BZ) was set as 21×21×1 for 2D graphene and silicene and 15×15×15 for 3D silicon, respectively. The second-order IFC and the third-order IFCs were recorded by the finite displacement method as implemented in the PHONOPY code [54] and thirdorder_vasp.py code [55], respectively. We built a 5×5×1 supercell for graphene and silicene and a 3×3×3 supercell for silicon to obtain the second-order IFCs. In order to ensure a large NN in graphene, when calculating the third-order IFCs, we used a 7×7×1 supercell so that a large $r_{c}$ up to the 15^th^ NN can be correctly modeled, while for another two materials, the 5×5×1 supercell and 4×4×4 supercell were used for silicene and silicon, respectively. We used a finite displacement of 0.01 Å in the PHONOPY package, which is sufficient to ensure a convergent dynamical matrix.

Previous studies revealed the important role of the four-phonon scattering process in determining lattice thermal conductivity [20,21,56]. However, due to the complexity in modeling four-phonon scattering, only a short cutoff distance up to the second nearest neighbors can be considered in the calculation of fourth-order interatomic force constants [57,58,59,60]. Since our objective was to study the effect of a long-range cutoff distance in computing thermal conductivity, we limited our study to only the three-phonon scattering process.

By iteratively solving the BTE in the framework of three-phonon scattering, the lattice thermal conductivity tensor $\kappa^{\alpha \beta }$ can be obtained as implemented in the ShengBTE package [55]:

(1) $$\kappa^{\alpha \beta }=\frac{1}{k_{B}T^{2}\Omega }\sum_{\lambda }f_{0}\left(f_{0}+1\right){\left(\hbar \omega_{\lambda }\right)}^{2}\upsilon_{\lambda }^{\alpha }F_{\lambda }^{\beta },$$

where λ, ℏ, ω, $k_{B}$, *T*, Ω, $f_{0}$, and υ denote the phonon mode index, reduced Planck constant, phonon frequency, Boltzmann constant, temperature, system volume, equilibrium Bose–Einstein distribution, and phonon group velocity, respectively. Here, α and β are the Cartesian coordinate directions and the term $F_{\lambda }^{\beta }$ denotes the linear coefficient of the nonequilibrium phonon distribution function given by

(2) $$F_{\lambda }^{\beta }=\tau_{\lambda }^{0}\left(\upsilon_{\lambda }^{\beta }+\Delta_{\lambda }^{\beta }\right),$$

where $\tau_{\lambda }^{0}$ is the phonon lifetime obtained under single-mode relaxation time approximation (RTA). The term $\Delta_{\lambda }^{\beta }$ is obtained by the fully iterative solution of the BTE, which is a correction term to reflect the phonon distribution deviation from the RTA scheme. In the calculations, one can start with the RTA solution and obtain $F_{\lambda }^{\beta }$ by iteratively solving the BTE.

Based on Matthiessen’s rule, the phonon lifetime $\tau_{\lambda }^{0}$ can be expressed as

(3) $$\frac{1}{\tau_{\lambda }^{0}}=\frac{1}{\tau_{\lambda }^{3ph}}+\frac{1}{\tau_{\lambda }^{iso}},$$

where $\frac{1}{\tau_{\lambda }^{iso}}$ is the isotope scattering rate and $\frac{1}{\tau_{\lambda }^{3ph}}\;$ is the three-phonon scattering rate. The intrinsic scattering rate $\frac{1}{\tau_{\lambda }^{3ph}}\;$ can be calculated by Fermi’s golden rule as

(4) $$\frac{1}{\tau_{\lambda }^{3ph}}=\frac{1}{N}\left(\sum_{\lambda_{1}\lambda_{2}}^{+}\Gamma_{\lambda \lambda_{1}\lambda_{2}}^{+}+\sum_{\lambda_{1}\lambda_{2}}^{-}\frac{1}{2}\Gamma_{\lambda \lambda_{1}\lambda_{2}}^{-}\right),$$

where *N* is the total number of *q* points and Γ with the superscripts (±) represents the scattering rates for three-phonon processes given by:

(5) $$\Gamma_{\lambda \lambda_{1}\lambda_{2}}^{+}=\frac{\hbar \pi }{4}\frac{\left(f_{0}^{\lambda_{1}}-f_{0}^{\lambda_{2}}\right)}{\omega_{\lambda }\omega_{\lambda_{1}}\omega_{\lambda_{2}}}{\left|V_{\lambda \lambda_{1}\lambda_{2}}^{+}\right|}^{2}\delta \left(\omega_{\lambda }+\omega_{\lambda_{1}}-\omega_{\lambda_{2}}\right),\;$$

(6) $$\Gamma_{\lambda \lambda_{1}\lambda_{2}}^{-}=\frac{\hbar \pi }{4}\frac{\left(f_{0}^{\lambda_{1}}+f_{0}^{\lambda_{2}}+1\right)}{\omega_{\lambda }\omega_{\lambda_{1}}\omega_{\lambda_{2}}}{\left|V_{\lambda \lambda_{1}\lambda_{2}}^{-}\right|}^{2}\delta \left(\omega_{\lambda }-\omega_{\lambda_{1}}-\omega_{\lambda_{2}}\right).\;$$

The scattering matrix $V_{\lambda \lambda_{1}\lambda_{2}}^{\pm }$ is given by

(7) $$V_{\lambda \lambda_{1}\lambda_{2}}^{\pm }=\sum_{i,j,k}\sum_{\alpha \beta \gamma }\Phi_{ijk}^{\alpha \beta \gamma }\frac{e_{\lambda }^{i\alpha }e_{\pm \lambda_{1}}^{j\beta }e_{-\lambda_{2}}^{k\gamma }}{\sqrt{m_{i}m_{j}m_{k}}},$$

where $\Phi_{ijk}^{\alpha \beta \gamma }$ is the third-order IFCs, *i*, *j*, and *k* are the atomic indices, *m* denotes the atomic mass, and $e_{\lambda }^{i\alpha }$ is the α^th^ component of the phonon eigenvector on atom *i*. More details about the BTE calculations can be found in our previous works [19,38].

## 3. Results and Discussion

**Figure 1:**
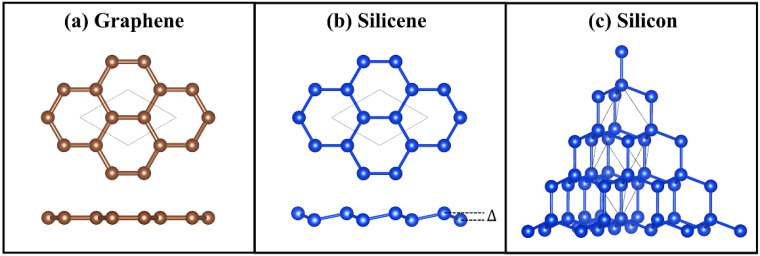
The atomic structure of (**a**) graphene, (**b**) silicene, and (**c**) bulk silicon. Graphene has an atomically flat surface, while the surface of silicene has a finite buckling height Δ.

The atomic structures of graphene and silicene are shown in Figure 1a,b, respectively. Although silicene has a similar 2D honeycomb structure as graphene, there exists a buckling height Δ = 0.45 Å in silicene. The calculated lattice constants for graphene and silicene are 2.47 Å and 3.86 Å, respectively, in good agreement with the literature results [41,43,61]. In comparison, Figure 1c shows the atomic structure of 3D bulk silicon, with a lattice constant of 5.47 Å, which also agrees well with the previous study [55]. The phonon dispersion (Figure S1 in the Supporting Information) reveals that the quadratic dispersion of the ZA mode is only observed in 2D graphene and silicene, while all acoustic branches are linear in 3D bulk silicon.

**Figure 2:**
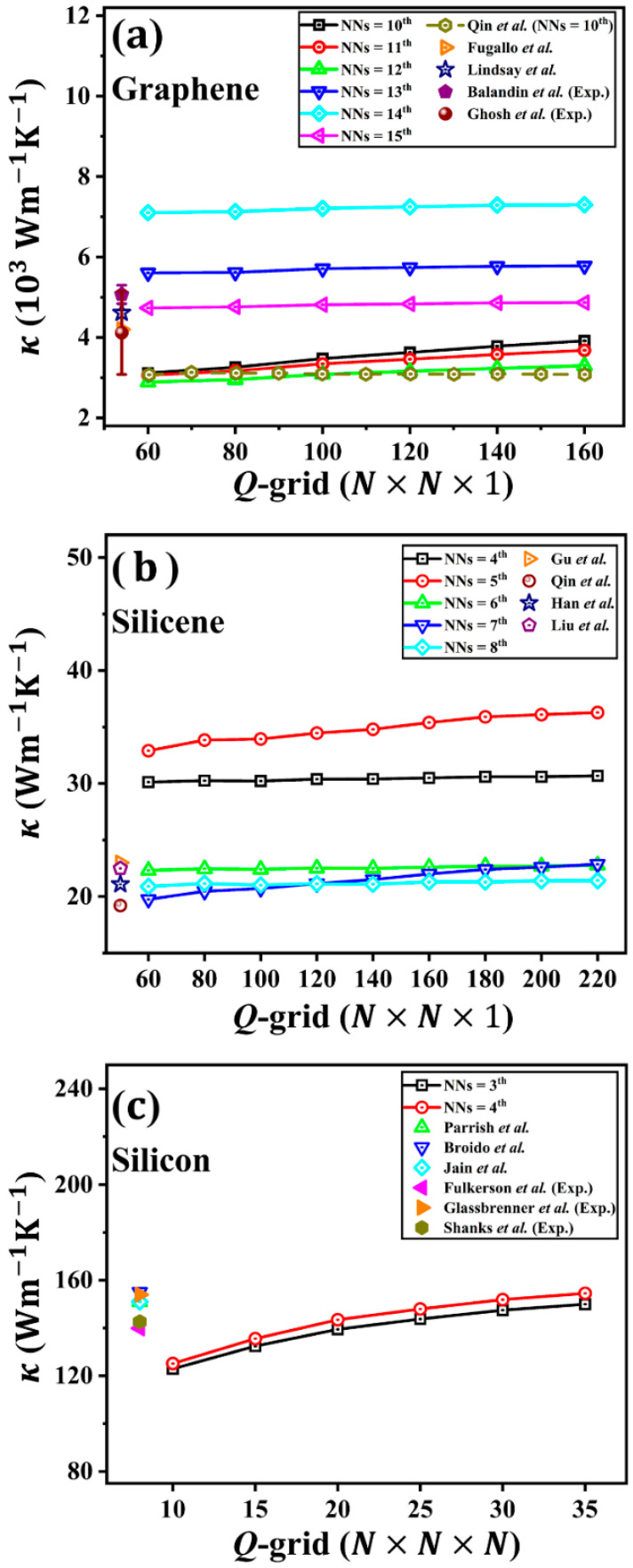
The room temperature thermal conductivity κ with respect to the *Q*-grid for different nearest neighbors (NNs). (**a**) Graphene. The literature results were adapted from Qin et al. [40], Fugallo et al. [62], Lindsay et al. [63], Balandin et al. [4], and Ghosh et al. [64]. (**b**) Silicene. The literature results were adapted from Gu et al. [65], Qin et al. [66], Han et al. [67], and Liu et al. [68]. (**c**) Bulk silicon. The literature results were adapted from Parrish et al. [69], Broido et al. [70], Jain et al. [39], Fulkerson et al. [71], Glassbrenner et al. [72], and Shanks et al. [73].

We first verified the calculated κ of graphene versus the *Q*-grid size (N×N×1). Since a 7×7×1 supercell was used for graphene in our calculations, the largest $r_{c}$ should be less than half of the length of the supercell due to the periodic boundary condition. This means the upper limit of $r_{c}$ is 8.645 Å, which corresponds to the 15^th^ NN ($r_{c}$ = 8.61 Å). The detailed relation between $r_{c}$ and the *n*^th^ NN can be found in Figure S2 in the Supporting Information. Figure 2a shows the convergence for the calculated κ of graphene at 300 K. When the cutoff radius is small (NNs = 10^th^, 11^th^, and 12^th^), the value of κ diverges with the increasing *Q*-grid. A similar divergent κ was also reported by Qin and Hu [40] when the $r_{c}$ is small. However, we noticed our calculation results are quantitatively different from the results of Qin and Hu [40], in which a converged κ for NNs = 10^th^ was reported by Qin and Hu’s work (empty hexagon in Figure 2a). A 5×5×1 supercell was used in their study, which sets the upper limit of $r_{c}$ around 6.17 Å. However, $r_{c}$ = 6.35 Å (NNs = 9^th^) and $r_{c}\;$= 6.80 Å (NNs = 10^th^) were used in their calculations. This discrepancy between our result and the previous study highlights the importance of using a sufficiently large supercell when increasing the cutoff radius.

When the $r_{c}$ further increases beyond the 12^th^ NN, a convergent κ with respect to the *Q*-grid size is observed in graphene. This is because the long-range interaction can affect the lifetime of low-frequency phonons, which correspond to acoustic phonon modes near the Γ point [40]. However, the converged κ values depend notably on the specific choice of $r_{c}$. For instance, the predicted κ at 300 K with the 13^th^, 14^th^, and 15^th^ NN is 5780 Wm^−1^K^−1^, 7300 Wm^−1^K^−1^, and 4867 Wm^−1^K^−1^, respectively, exhibiting a non-monotonic dependence on $r_{c}$. A further increase of $r_{c}$ is not possible unless an even larger supercell than 7×7×1 is used, which is beyond our computational capacity. As shown in Figure 2a, the predicted κ value for the largest $r_{c}$ considered in this study (NNs = 15^th^) agrees reasonably well with the previous theoretical and experimental results [4,62,63,64]. This complex convergence behavior in graphene is intriguing and also demonstrates that the cutoff radius is very important in determining the κ of graphene.

As another 2D material, the non-monotonic dependence of κ on $r_{c}$ is also observed in silicene, as shown in Figure 2b. However, when the $r_{c}$ is beyond the 6^th^ NN, the predicted κ value at 300 K in silicene reaches convergence much faster compared to that in graphene. In silicene, the converged κ at the large *Q*-grid limit for the 6^th^, 7^th^, and 8^th^ NN is 22.77 Wm^−1^K^−1^, 22.85 Wm^−1^K^−1^, and 21.42 Wm^−1^K^−1^, respectively. These values agree quite well with the results recorded in the literature [65,66,67,68]. In contrast, when switching to a 3D material, this significant convergence issue with respect to $r_{c}$ observed in the 2D material vanishes in the bulk silicon (Figure 2c). In the large *Q*-grid limit, the predicted κ value at 300 K in silicon for the 3^rd^ and 4^th^ NN is 150 Wm^−1^K^−1^ and 154 Wm^−1^K^−1^, respectively, which is also in accordance with the literature results [39,69,70,71,72,73]. By comparing these three materials, we found that the κ of the 3D material converges more easily than the 2D material, while the κ of graphene is more difficult to converge in the 2D material. This difference in the convergence of κ in the above-mentioned three materials aroused our interest in the intrinsic phonon transport form.

The different convergence behaviors of κ between the 2D and 3D materials remind us of the unique ZA branches in 2D materials. As a special feature of 2D materials, the flexural ZA phonons not only lead to unique phonon transport regimes, but also facilitate the momentum-conserving normal phonon–phonon scattering (N-scattering) [30]. When the N-scattering dominates over other phonon scatterings, such as Umklapp scattering (U-scattering), hydrodynamic phonon transport will take place [27,29].

In order to quantitatively compare the degree of hydrodynamic phonon transport among these three materials, we computed the ratio between two thermal conductivity predictions, i.e., the iterative solution ($\kappa_{I}$) and relaxation time approximation ($\kappa_{\mathrm{RTA}}$). The relaxation time approximation (RTA) incorrectly treats the N-scattering as a resistive process, the same as U-scattering. As a result, RTA overestimates the phonon–phonon scattering rates and, thus, leads to an underestimation of κ compared to the iterative solution, especially when the N-scattering is dominant [26,28]. Therefore, the difference between $\kappa_{I}$ and $\kappa_{\mathrm{RTA}}$ can serve as an indicator of hydrodynamic phonon transport.

**Figure 3:**
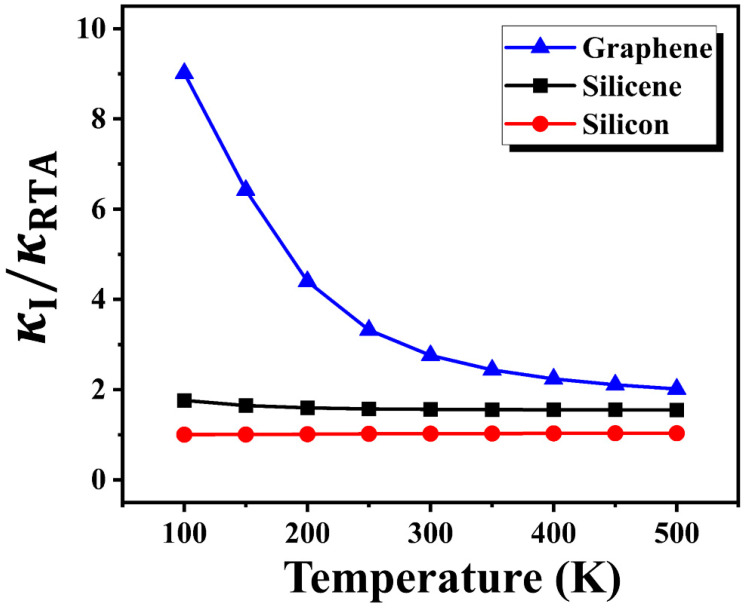
Thermal conductivities from the iterative solution ($\kappa_{I}$) and relaxation time approximation ($\kappa_{\mathrm{RTA}}$) of the BTE for graphene, silicene, and silicon.

Figure 3 shows the ratio $\kappa_{I}$/$\kappa_{\mathrm{RTA}}$ versus temperature for three materials. For both graphene and silicene, this ratio is larger than unity, indicating the emergence of hydrodynamic phonon transport in these 2D materials. With decreasing temperature, this ratio gradually increases in both graphene and silicene, due to the suppressed U-scattering at a low temperature. Moreover, this ratio in graphene is larger than that in silicene at a given temperature, and the decreasing trend of this ratio with decreasing temperature is much more obvious in graphene. These results suggest a stronger hydrodynamic behavior occurring in graphene. In contrast, this ratio in bulk silicon is close to unity and is insensitive to temperature, confirming that there is no hydrodynamic phonon transport in 3D silicon.

**Figure 4:**
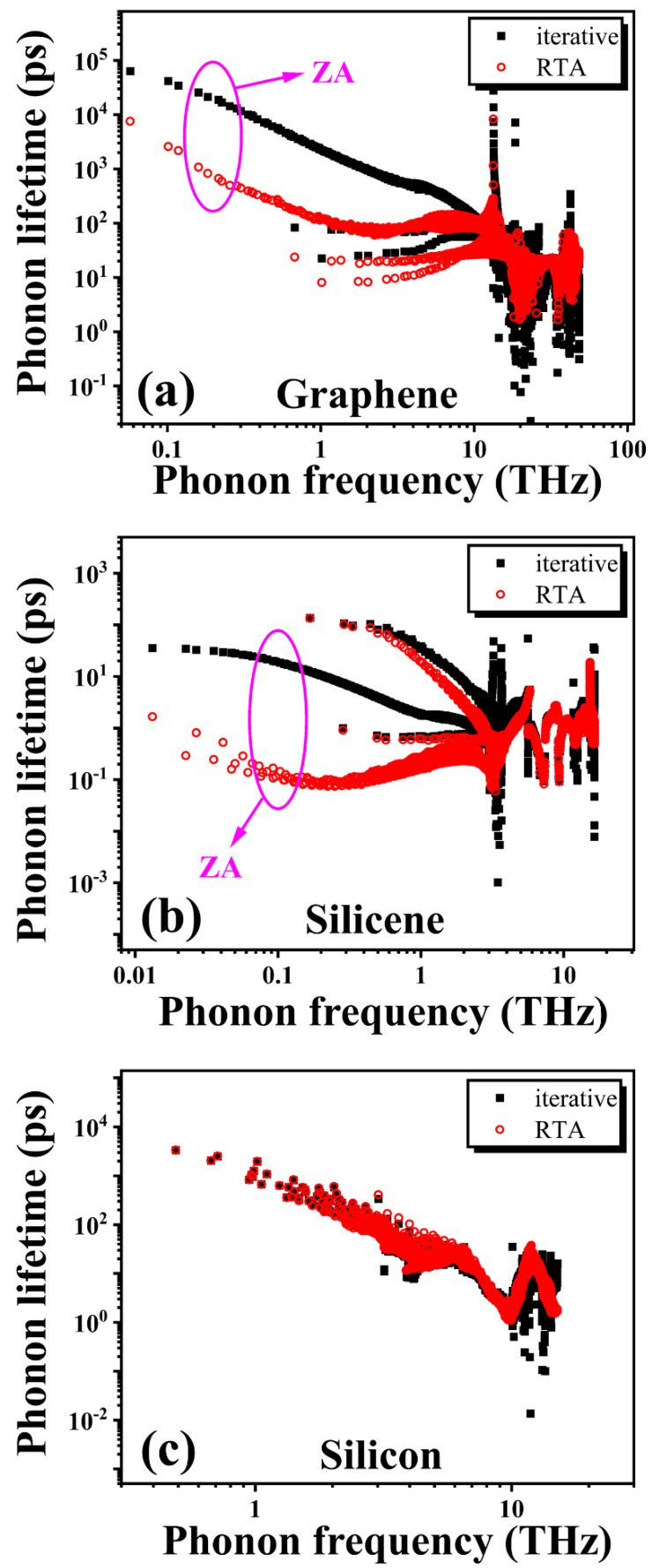
The phonon lifetime calculated from the iterative solution and relaxation time approximation at 300 K in (**a**) graphene, (**b**) silicene, and (**c**) bulk silicon. Huge differences in the phonon lifetime for the low-frequency ZA mode are observed in graphene and silicene.

In order to understand the origin for the different degrees of hydrodynamic phonon transport, we further calculated the phonon lifetime from both iterative and RTA solutions for these three materials. In the 2D system, there exists notable differences in the phonon lifetime between the iterative and RTA solutions for both graphene (Figure 4a) and silicene (Figure 4b), especially for the low-frequency phonons near the Γ point. These low-frequency phonons actually correspond to the ZA branch. Moreover, graphene has a mirror reflection symmetry due to the atomically flat surface, while silicene has a buckled surface. The mirror reflection symmetry in graphene imposes a *symmetry selection rule* on ZA phonons, causing the three-phonon processes involving an odd number of flexural phonons to be forbidden [63,74,75]. This unique selection rule in graphene gives rise to a surprisingly large phonon lifetime for ZA phonons near the Γ point, which is larger than that in silicene by more than three-orders of magnitude. In contrast, there exists a negligible difference in the phonon lifetime between the iterative and RTA solutions for the bulk silicon (Figure 4c). These results reveal that the ZA phonon is responsible for the hydrodynamic phonon transport observed in 2D graphene and silicene.

We demonstrated the convergence issue of κ and hydrodynamic phonon transport in 2D materials, while the same phenomenon is absent in 3D materials. In order to understand the physical origin for this difference, we finally computed the frequency-resolved phonon scattering rate from Fermi’s golden rule for both N-scattering ($\Gamma_{N}\left(\omega \right)$) and U-scattering ($\Gamma_{U}\left(\omega \right)$) in these three materials at 300 K. To further compare the ensemble-averaged scattering strength, the averaged scattering rate is computed as [27,28,29]

(8) $${\bar{\Gamma }}_{s}=\frac{\sum C\left(\omega \right)\Gamma_{s}\left(\omega \right)}{\sum C\left(\omega \right)},$$

where the subscripts denote the N- or U-scattering and C(ω) is the mode specific heat given by

(9) $$C\left(\omega \right)=f_{0}\left(\omega \right)\frac{\left[f_{0}\left(\omega \right)+1\right]{\left(\hbar \omega \right)}^{2}}{k_{B}T^{2}}$$

**Figure 5:**
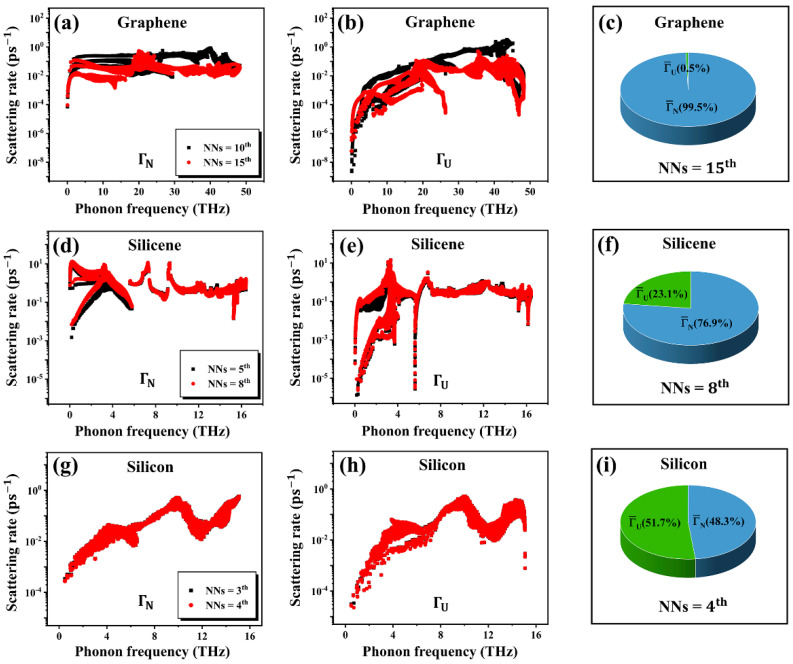
The frequency-resolved N-scattering rate ($\Gamma_{N}\left(\omega \right)$) and U-scattering rate ($\Gamma_{U}\left(\omega \right)$) at 300 K with different NNs in (**a**,**b**) graphene, (**d**,**e**) silicene, and (**g**,**h**) silicon. The ensemble-averaged scattering rates (${\bar{\Gamma }}_{N}$ and ${\bar{\Gamma }}_{U}$) for three acoustic phonons are shown in (**c**) graphene, (**f**) silicene, and (**i**) silicon, respectively.

Figure 5a,b show the computed $\Gamma_{N}\left(\omega \right)$ and $\Gamma_{U}\left(\omega \right)$ with different NNs in graphene, respectively. For a given NN, the $\Gamma_{N}\left(\omega \right)$ is much larger than $\Gamma_{U}\left(\omega \right)$ by two-orders of magnitude in the low-frequency region, which makes the most contribution to the thermal conductivity. The ensemble-averaged scattering rate for the acoustic phonons is ${\bar{\Gamma }}_{N}\;$= 2.843 ${\mathrm{ps}}^{-1}$ and ${\bar{\Gamma }}_{U}\;$= 0.015 ${\mathrm{ps}}^{-1}$. In other words, ${\bar{\Gamma }}_{N}$ accounts for 99.5% of the total scatterings, demonstrating the dominance of N-scattering over U-scattering. This gives rise to the hydrodynamic phonon transport in graphene, as witnessed in Figure 4. In the hydrodynamic transport regime, phonons experience little scattering inside the material and, thus, can propagate collectively, which leads to very long phonon mean free path (MFP) and fast transport of thermal energy [27]. Such a long MFP and collective motion of phonons result in a long-range interaction between atoms in graphene. This effect can be revealed by the obvious dependence of $\Gamma_{N}\left(\omega \right)$ and $\Gamma_{U}\left(\omega \right)$ on the cutoff radius (NNs). Therefore, the hydrodynamic-transport-induced long-range interaction is responsible for the significant convergence issue of κ for graphene observed in Figure 1a.

Similar to graphene, Figure 5d,e reveal that the $\Gamma_{N}\left(\omega \right)$ in silicene is also larger than $\Gamma_{U}\left(\omega \right)$ in the low-frequency region, and ensemble-averaged scattering rate ${\bar{\Gamma }}_{N}$ accounts for 76.9% of the total scatterings, which is larger than ${\bar{\Gamma }}_{U}$. However, the difference between ${\bar{\Gamma }}_{N}$ and ${\bar{\Gamma }}_{U}$ in silicene is much smaller than that in graphene. Furthermore, the scattering rate spectrum in silicene is less sensitive to the choice of NNs compared to that in graphene. These results suggest a weaker degree of hydrodynamic phonon transport in silicene compared to graphene. This in turn leads to a faster convergence of κ at a smaller NN (Figure 2b) and a smaller difference in κ between the two BTE solutions (Figure 4) in silicene, compared to the case of graphene. In contrast, when it shifts to a 3D material, Figure 5g,h show no difference in the phonon scattering rate spectrum between different NNs in the bulk silicon, and the averaged scattering rate in Figure 5i confirms that the U-scattering is the dominant scattering process. This results in the vanishing of hydrodynamic phonon transport (Figure 4) and, consequently, no convergence issue of κ for the bulk silicon, even at very small NNs (Figure 2c).

## 4. Conclusions

In summary, we calculated the room temperature thermal conductivity κ of graphene, silicene, and bulk silicon within different cutoff radius $r_{c}$. In contrast to the fast convergence of κ at very small $r_{c}$ in the 3D bulk silicon, the κ of the 2D materials exhibits a persistent dependence on $r_{c}$ in both graphene and silicene, which is more pronounced in graphene even up to the 15^th^ nearest neighbor. By comparing the κ values predicted from the iterative solution and relaxation time approximation, the hydrodynamic phonon transport was confirmed in both graphene and silicene, while it is absent in the bulk silicon. More interestingly, the sensitivity of κ with $r_{c}$ in these three materials correlates very well with the strength of the hydrodynamic transport behavior. The calculations of phonon lifetime revealed that the presence of a unique ZA phonon in 2D materials is a key factor for the hydrodynamic phonon transport observed in graphene and silicene. By further analyzing the scattering rates, we demonstrated that the dominance of the N-scattering process causes the hydrodynamic transport, so that phonons can propagate collectively, which results in a long-range interaction in a 2D material such as graphene compared to the 3D material. Therefore, the long-range interaction induced by the strong hydrodynamic phonon transport is responsible for the significant convergence behavior of κ observed in graphene. This work uncovers the underlying physical mechanism for the notable convergence behavior of κ in 2D systems.

## Data Availability

The data presented in this study are available from the corresponding author upon reasonable request.

## Supplementary Materials

The following supporting information can be downloaded at: https://www.mdpi.com/article/10.3390/nano12162854/s1, Figure S1: The phonon dispersion of graphene, silicene, and silicon. Figure S2: The corresponding cutoff radius ($r_{c}$) to the nth nearest neighbors (NNs) for graphene, silicene, and silicon.

## Nomenclature

κ	thermal conductivity
$r_{c}$	cutoff radius
NN	nearest neighbor
ℏ	reduced Planck constant
ω	phonon frequency
$k_{B}$	Boltzmann constant
*T*	temperature
Ω	system volume
$f_{0}$	equilibrium Bose–Einstein distribution
υ	phonon group velocity
$\tau_{\lambda }^{0}$	phonon lifetime
C(ω)	mode specific heat
α, β	Cartesian coordinate directions
*m*	atomic mass
*i*, *j*, *k*	atomic indices
$\kappa_{I}$	thermal conductivities from the iterative solution
$\kappa_{\mathrm{RTA}}$	thermal conductivities from the relaxation time approximation
$\Gamma_{N}$	normal scattering rates
$\Gamma_{U}$	Umklapp scattering rates
${\bar{\Gamma }}_{N}$	averaged normal scattering rates
${\bar{\Gamma }}_{U}$	averaged Umklapp scattering rates
