# Supplementary material for: How Hydrodynamic Phonon Transport Determines the Convergence of Thermal Conductivity in Two-Dimensional Materials
Source: Nanomaterials (Basel). 2022 Aug 18;12(16):2854. doi: 10.3390/nano12162854 (PMC9415093; doi:10.3390/nano12162854)
Supplement: Supplementary file 1 [file nanomaterials-12-02854-s001.zip › nanomaterials-1853065-supplementary.pdf]

nanomaterials

Supporting Information for

How Hydrodynamic Phonon Transport Determines the Convergence of Thermal Conductivity in Two-Dimensional Materials

Jianhui Jiang, Shuang Lu, Yulou Ouyang and Jie Chen *

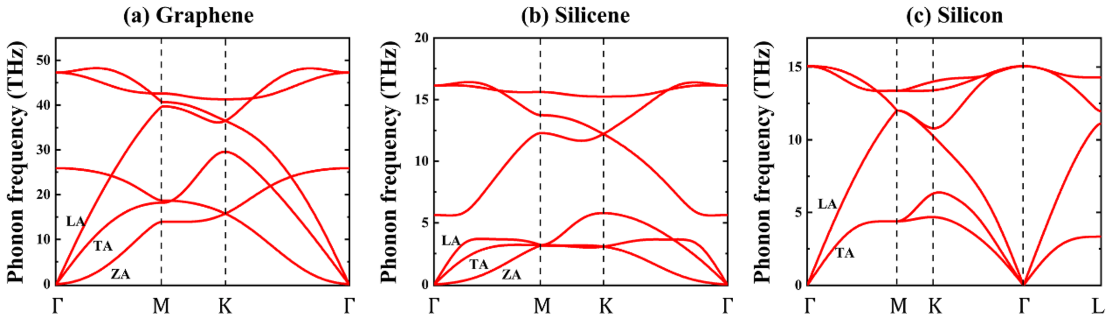

Figure S1. The phonon dispersion of (**a**) graphene, (**b**) silicene, (**c**) silicon. ZA, TA and LA branches are marked. The quadratic dispersion of ZA mode is only observed in 2D system.

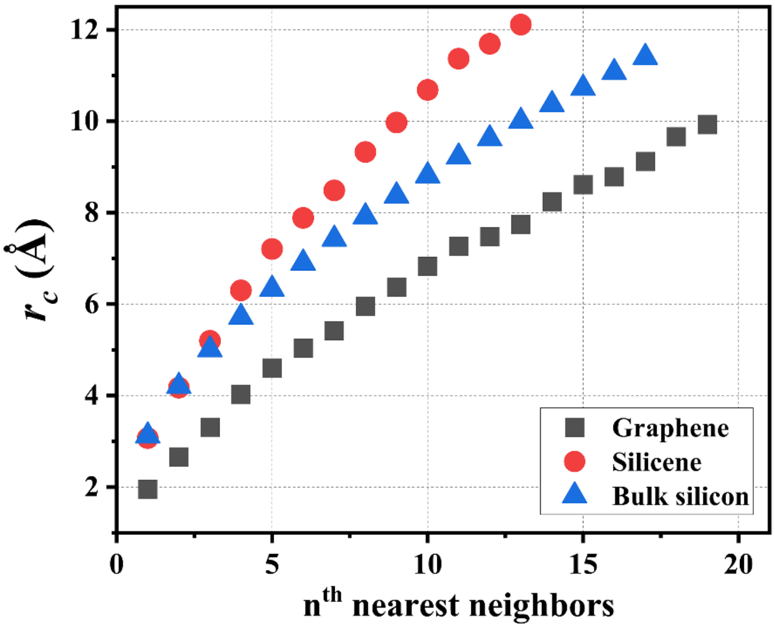

Figure S2. The corresponding cutoff radius (r_c) to the nth nearest neighbors (NNs) for graphene, silicene, and bulk silicon.
